# Object-based attention in complex, naturalistic auditory streams

**DOI:** 10.1038/s41598-019-39166-6

**Published:** 2019-02-27

**Authors:** Giorgio Marinato, Daniel Baldauf

**Affiliations:** Center for Mind/Brain Sciences (CIMeC), University of Trento, Trento, Italy

## Abstract

In vision, *objects* have been described as the ‘units’ on which non-spatial attention operates in many natural settings. Here, we test the idea of object-based attention in the auditory domain within ecologically valid auditory scenes, composed of two spatially and temporally overlapping sound streams (speech signal vs. environmental soundscapes in Experiment 1 and two speech signals in Experiment 2). Top-down attention was directed to one or the other auditory stream by a non-spatial cue. To test for high-level, object-based attention effects we introduce an auditory *repetition detection task* in which participants have to detect brief repetitions of auditory objects, ruling out any possible confounds with spatial or feature-based attention. The participants’ responses were significantly faster and more accurate in the valid cue condition compared to the invalid cue condition, indicating a robust cue-validity effect of high-level, object-based auditory attention.

## Introduction

In many ecologic environments, the naturalistic auditory scene is composed of several concurrent sounds with their spectral features overlapping both in space and time. Humans can identify and differentiate overlapping auditory objects surprisingly well^1^. This ability was first described in the literature in form of the “cocktail party problem”^2^, which is still one of the most successful paradigms in research on auditory perception. The original term was introduced to describe the particular situation of a multi-talker environment, like a cocktail party, in which a person has to select a particular speech signal, filtering out other, distracting sound signals. The challenge in such a cocktail party situation is due to the fact that all the sounds in the auditory scene, sum together linearly into one single sound stream per ear. Only by segregating features originating from different spatial sources and by grouping together features originating from the same spatial source a listener can individuate the intended sound stream and then parse out the respective auditory objects from the mixture of the scene. The mechanism by which the single signal is segregated in different sound objects was termed *sound segregation* or “auditory scene analysis”^3^. According to McDermott^4^, the identification of different sounds in a complex auditory scene is mainly studied from two conceptually distinct perspectives: sound segregation (or “auditory scene analysis”)^3^ and attentional selection (first introduced by Cherry)^2^. According to the biased competition model^5^ selective attention is the central mechanism that biases processing for behaviorally relevant stimuli by facilitating the processing of important information and at the same time filtering out or suppressing irrelevant information.

Auditory research has focused primarily on the segregation component^6–9^, and despite of many efforts to better understand the interaction between auditory attention and segregation processes^8,10–15^, there is still debate about the mechanisms of auditory object formation and auditory selective attention^16–20^. However, attentional mechanisms have been described in much detail in other sensory modalities, in particular, in vision. From visual attention research we have learnt how top-down attentional control can operate on visual space^21–29^, οn low-level perceptual features^30–39^, and high-level visual objects^40–47^.

And especially visual *objects* have been described as the ‘units’ on which non-spatial attention operates in many natural settings^43,45,48^. In the auditory domain, we know much less about how selective attention can operate in a non-spatial manner. Particularly, we lack a better understanding of how attention can facilitate *object* units^49^, and guide selection on the level of segregated sounds. Such interaction of auditory selective attention and sound segregation remains an open issue^4^, preventing a more comprehensive understanding of both the cocktail party phenomenon and auditory scene analysis.

Within the domain of auditory selective attention, the experimental work that explicitly tried to tackle the interaction between top-down object-based attention and auditory scene analysis is relatively small in comparison to experimental work on the stimulus-based psychophysics of sound perception. Early work exploited mainly the “dichotic listening” paradigm^2^. In this paradigm, participants listen to a different audio stream presented to each ear and are asked to pay attention to either one of them^50–52^, or sometimes to both^53–56^. However, the dichotic listening paradigm always have a spatial component to them and therefore leave plenty of room for attentional lateralization confounds, which constitute a major shortcoming for using them to investigate non-spatial attention. Later work used paradigms that manipulated specific features of the acoustic stimulus to demonstrate successful tracking of one sound signal over the other. Some studies modulated pitch^19,57^, others intensity level^58^ or spatial features, such as location^19^. More recent studies, focused on the mechanisms of the neural representation of speech signals, using neural recordings for precisely tracking speech signals^59–62^. Lastly high-level attention modulation in a complex auditory scene was investigated from the neural perspective also with paradigms that involve competing speech streams^63,64^, speech in noise^58^, and tone streams^65,66^.

Here, we introduce a novel stimulus set and task to study object-based attention in the auditory domain. In analogy to visual objects, we defined an *auditory object* as the aggregation of low-level features into grouped entities. Several auditory objects together can then constitute an auditory scene, or soundscape, e.g. the characteristic soundscape of a railroad station or a multi-talker conversation at a party. In such natural, complex auditory environments, auditory objects are temporally confined and bound entities, e.g., the words constituting a conversation or a train whistle in the soundscape of a railroad station. Notably, there have already previously been various attempts to define what an *auditory object* is, e.g., by exploring the rules of its formation from a background of competing sounds, but without reaching yet an unanimous consensus on how the diverse mechanisms work together^1,3,17^. One influential operational definition was proposed by Griffiths and Warren^1^. Here, an auditory object is defined as something (1) that corresponds to things in the sensory world, (2) that can be isolated from the rest of the sensory world, and (3) that can be recognized or generalized beyond the single particular sensory experience. Further, object analysis may also involve generalization across different sensory modalities, such as the correspondence between the auditory and visual domain^1^. This operational definition has also been used to define the neural representation of auditory objects^8^. Our definition borrows from the previous ones and is in line with the concept of acoustic stream, or ‘soundscape’, as a superordinate entity of individual objects^67^.

Again in analogy to visual paradigms used to study object-based attention^68^, we introduce an auditory repetition detection task, in which participants had to detect brief repetitions of auditory objects within the acoustic stream of a soundscape. The logic behind this new task is that such a repetition detection task requires the participants to fully process the acoustic stream to a cognitive level that allows them to recognize a certain, temporally extended set of low-level features as an object and to understand that this set of features was repeated. Importantly, this attention task cannot be solved by attending to a distinct low-level *feature* itself (e.g., a certain pitch). To also rule-out *spatial attention*, we presented two overlapping auditory scenes (e.g., in Experiment 1 a foreign language conversation and a railroad station soundscape) at the same external speaker, attentionally cuing one or the other.

In every trial, a 750 ms long repetition is introduced in one of the two overlapping streams and participants are asked to detect any such repetitions of auditory objects as fast as possible. This task requires the processing of the acoustic stream to the level of auditory objects and is specifically designed to investigate object-based mechanism of selective attention, i.e., whether top-down selective attention can weigh incoming acoustic information on the level of segregated auditory objects by facilitation and/or inhibition processes.

## Experiment 1: Attentional Weighting of Speech Versus Environmental Soundscenes

### Methods

#### Participants

Eleven participants (6 females, 5males, mean age 25.7 years, range 23–32 years, all of them right-handed and normal hearing) took part in the behavioral experiment and were paid for their participation. All participants were naïve in respect to the purpose of the study and they were not familiar with any of the languages used to create the speech stimuli. All participants provided written, informed consent in accordance with the University of Trento Ethical Committee on the Use of Humans as Experimental Subjects. One participant had to be excluded from further analyses because he failed to follow the task instructions.

#### Stimuli

Speech and environmental sound signals: The experimental stimuli were auditory scenes, consisting of overlapping streams of (a) speech conversations embedded in (b) environmental sounds. All the speech signals were extracted from newscast recordings of various foreign languages: (1) African dialect, (2) Amharic, (3) Armenian, (4) Bihari, (5) Hindi, (6) Japanese, (7) Kurdish, (8) Pashto, (9) Sudanese, (10) Urdu, (11) Basque, (12) Croatian, (13) Estonian, (14) Finnish, (15) Hungarian, (16) Icelandic, (17) Macedonian, (18), Mongolian, and (19) Bulgarian. The environmental sound source signals were selected from soundscapes of public human places, recorded at (1) airports, (2) canteens, (3) malls, (4) markets, (5) railway stations, (6) restaurants, (7) streets, (8) trains, and (9) subways.

From each recording, we extracted a central part using Audacity software, discarding the very beginning and end of the original signal. All recording segments were processed with Matlab custom functions to cut the sound segments to 5 seconds length, convert them to mono by averaging both channels, and normalize them to -23db.

Guided by the Urban Sound Taxonomy^69^ and Google’s Audio Set^70^ we chose the stimuli from high quality YouTube recordings.

Enveloping: After these processing steps, speech signals and environmental signals still differed in their low-frequency rhythmicity and overall signal envelope: the analytical envelopes of the environmental sound epochs were rather stationary whereas speech signals are characterized by prominent quasi-rhythmic envelope modulations in the 4–8 Hz range. In order to further equalize the two sound streams and make them as comparable as possible we dynamically modulated the envelope of the environmental sounds using envelopes randomly extracted from the speech signals. To do so envelopes of the speech signals were first extracted using the ‘Envelope’ functionality in Matlab, which is based on the spline interpolation over local maxima separated by at least 4410 samples, corresponding to 0.1 s at a sample rate of 44.1KHz. This relative large number of samples was chosen in order to keep the environment sound clearly recognizable after applying a quasi-rhythmic temporal.

**Figure 1:**
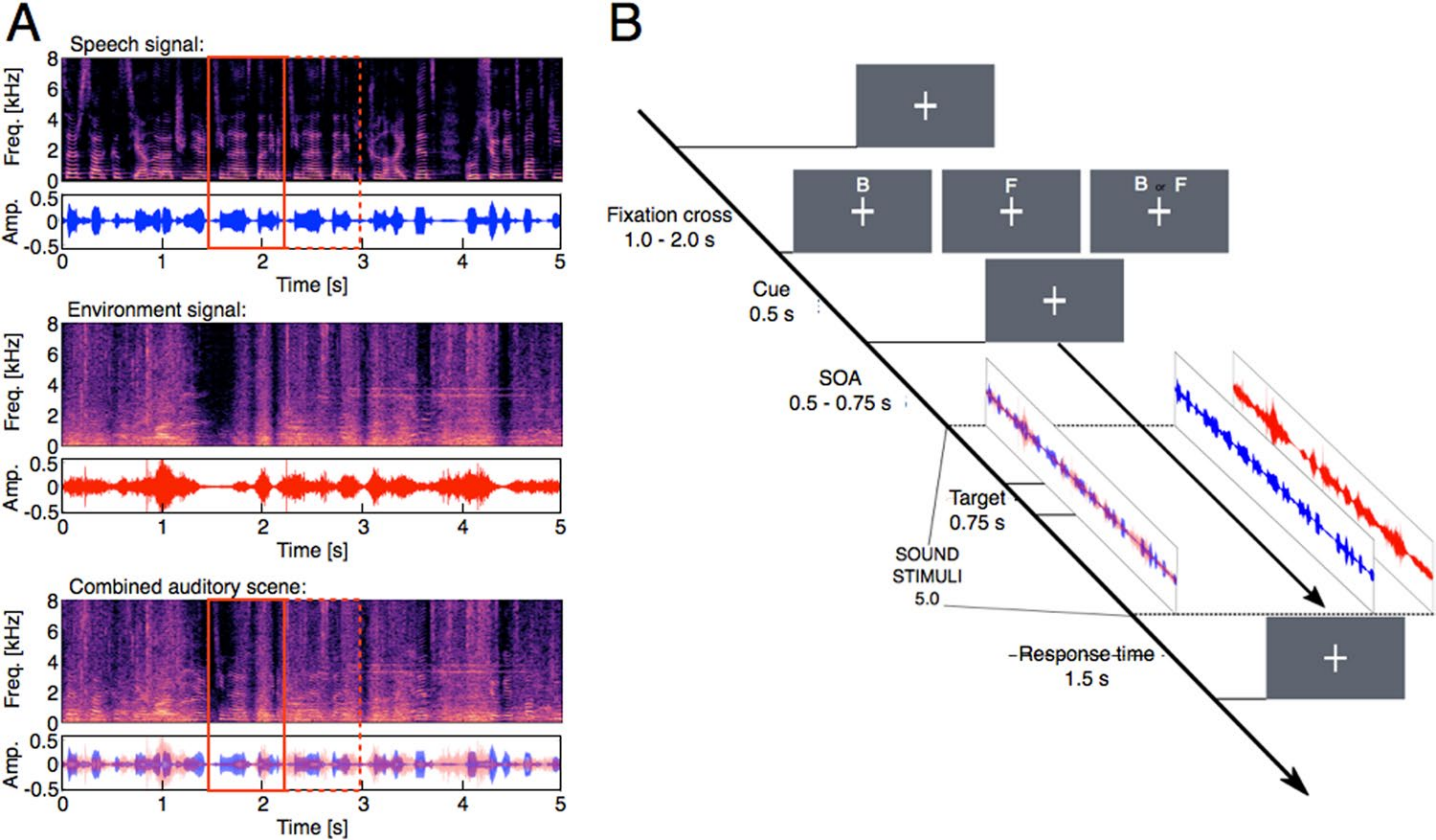
(**A**) Experimental acoustic stimuli. In each trial, the acoustic stimuli consisted of two soundscapes, one foreign language speech signal and one environmental sound signal (e.g., the soundscape of a train station), which were temporally and spatially overlaid, presented from the same centrally positioned speaker for a total of 5 seconds. The three subpanels show the time-frequency spectrogram and raw amplitude spectra of examples of the original sound streams (i.e., speech signal and the environmental signal, respectively), as well as for the combined auditory scene that was presented to the participants (lower panel). In one of the two streams (here in the speech signal), a repetition target was embedded by replicating a 750-ms interval (see the solid red box) and repeating it directly after the original segment (see the dashed red box). Linear ramping and cross-fading algorithms were applied to avoid cutting artifacts and to render the transition between segments unnoticeable. The repetition targets had to be detected as fast and as accurate as possible. (**B**) Sequence of a typical trial. In each trial, a cue was presented indicating either the speech component of the signal (‘F’) or the environmental component (‘B’) or both (neutral cueing condition). Subjects were instructed to shift their attention to the cued channel and to detect any repetition targets as fast and as accurate as possible, while keeping central eye fixation throughout the trial. Cue validity was 70%.

One-back repetition targets and overlay: In a next step, we inserted small segment repetitions to be used as repetition targets in our listening task (see Fig. 1A). For this we randomly sampled and extracted short sound epoch of 750 ms and repeated it immediately after the end of the segment that has been sampled. The length of the repetition targets was chosen to roughly correspond to a functional unit like a typical acoustic event in the environment sounds or a couple of syllables/words in the speech signals. In order to implement the repetition in Matlab, the initial sound signal was cut at a randomly selected sample, then the original beginning, the 750 ms repetition, and the original end to the stream were all concatenated by a linear ramping and cross-fading. The linear ramping is made by a window of 220 samples that corresponds to 5 ms at a sample rate of 44.1 KHz. The cross-fading is achieved by simply adding together the ramping down part of the previous segment with the ramping up part of the subsequent segment.

Finally, for each trial’s audio presentation one resulting speech signal and one environmental sound signal were overlapped to form an auditory scene, consisting of speech conversation embedded in environmental sounds (see Fig. 1A bottom panel). In each trial only one of them could contain a repetition target. A set of the experimental stimuli can be freely downloaded at 10.5281/zenodo.1491058.

Trial Sequence and Experimental Design: All stimuli were presented using Psychophysics Toolbox Version 3^71^. Figure 1B provides an overview of a typical trial sequence. We implemented an attentional cueing paradigm with three cue validity conditions, i.e. valid, neutral, and invalid cues. Cue validity was 70%, 20% of cues were invalid, and 10% neutral. At the beginning of each trial, a fixation-cross appeared and subjects were instructed to keep central eye fixation throughout the trial (see Fig. 1B). After an interval of 1.0–2.0 s (randomly jittered) a visual cue was presented, directing auditory attention either to the “Speech” signal stream or to the auditory “Environment” stream, or to neither of them in the neutral condition. After another interval of 0.5–0.75 s (randomly jittered) the combined audio scene with overlapping speech and environmental sounds started playing for 5.0 s. The participants were instructed to pay attention to the cued stream and to respond with a button press as soon as they recognize any repetition in the sound stimuli. Accuracy and speed were equally emphasized during the instruction.

Before the actual data collection, participants were first familiarized with the sound scenes and had a chance to practice their responses to repetitions for one blocks of 100 trials. For practice purposes, we initially presented only one of the two sound streams individually so participants had an easier time understanding what repetition signals to watch out for. This training lasted for 17 minutes in total.

Each subsequent testing block consisted of 100 trials but now with overlapping sound scenes consisting of both a speech and an environmental sound stream and with the described attentional cueing paradigm. Each participant performed 3 experimental blocks, resulting in 300 experimental trials in total. Overall our experimental design had two factors: (1) *Cue validity* with the conditions valid (70% of trials), neutral (10% of trials) and invalid (20% of trials), and (2) *Position of the repetition target* in either the speech (50% of trials) or environmental (50% of trials) sound stream. All conditions were trial-wise intermixed.

Data Analysis: All data analyses were performed with custom scripts in MATLAB. A combination of built-in function and custom code was used in order to conduct descriptive and inferential statistics. For each condition in our 2 × 3 factor, mean and standard error of the mean (SEM) were calculated both for reaction times and response accuracies. Repeated-measurement analyses of variance were computed on accuracy data, mean reaction times, signal detection sensitivity and response biases. To further investigate systematic differences between individual conditions we computed planned contrasts in form of paired-samples t-tests between the repetition detection rates and reaction times in the valid versus invalid versus neutral cueing condition (both in the speech and environmental sound stream).

However, differences in detection accuracy reaction times can also result from changes in the response bias, for example, by a tendency to reduce the amount of evidence that is required to decide whether a target had occurred. To better understand the stage of selection, i.e., whether increases in detection rate are due to changes in sensitivity or changes in the decision criterion, or both, we further computed signal detection theory (SDT) indices in form of the sensitivity indices (d’) and response bias or criterion (c).

### Results

#### Accuracy

**Figure 2:**
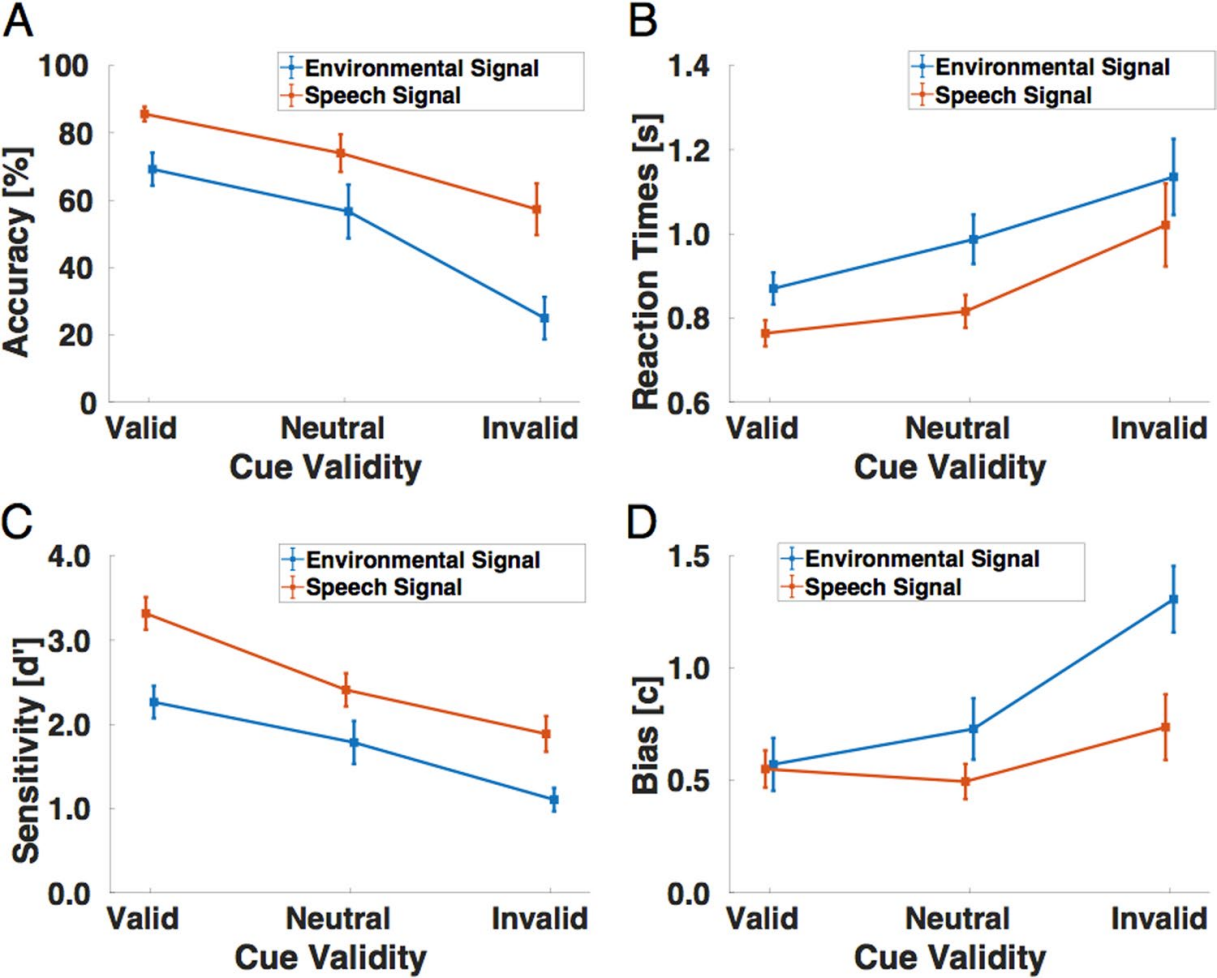
Experimental results in the repetition detection task of Experiment 1 with overlaid speech and environmental-noise streams. (**A**) Detection performances (in percent correct) as a function of cue validity (valid, neutral and invalid cueing condition), separately for both components of the acoustic stimuli, i.e., the environmental signal (blue) and the speech signal (red). The data are shown as means and SEM. (**B**) Reaction times (in seconds) as a function of cue validity (valid, neutral and invalid cueing condition), separately for both components of the acoustic stimuli (blue: environmental signal, red: speech signal). The data are shown as means and SEM. **(C)** Sensitivity scores (d’) and decision criteria **(D)** as a function of cue validity (valid, neutral and invalid cueing condition), separately for both components of the acoustic stimuli, i.e., the environmental signal (blue) and the speech signal (red). The data are shown as means and SEM.

Figure 2A shows the average accuracy with which repetition targets were detected in both the speech and environmental sound stream, and Fig. 2B shows the corresponding reaction times with which the responses were given. Repetition targets were detected well above chance, but performance was clearly not ceiling with up to 85% correct responses in the valid cueing condition. In general, valid cues helped the participants detecting repetition targets and also speeded up their responses by about 100 ms in respect to the neutral cueing condition. Invalid cues had the opposite effect. Table 1 provides an overview of the numeric values of mean detection accuracy and reaction times.

**Table 1:** Experiment 1 with overlaid speech and environmental sound streams.

	Valid	Neutral	Invalid
*Accuracy [%]*			
Speech	85.62 (2.17)	74.0 (5.57)	57.33 (7.66)
Environment	69.24 (4.87)	56.67 (7.97)	25.0 (6.27)
*Reaction time [ms]*			
Speech	764 (31)	816 (39)	1021 (98)
Environment	870 (38)	987 (59)	1136 (90)
*Sensitivity index (d’)*			
Speech	3.32 (0.19)	2.41 (0.20)	1.89 (0.21)
Environment	2.27 (0.19)	1.79 (0.25)	1.11 (0.14)
*Decision criterion (c)*			
Speech	0.55 (0.08)	0.50 (0.08)	0.74 (0.15)
Environment	0.57 (0.12)	0.73 (0.14)	1.31 (0.15)

Numeric values of the detection accuracy (in percent correct), reaction time (in ms), sensitivity indices (d’), and decision criteria (c), all across all ten participants for all three cueing conditions (valid, neutral and invalid cues), separately for the speech and environmental component of the signal. Values represent the means and standard errors of the mean.

A two-way repeated-measures analysis of variance (ANOVA) on the mean detection accuracy statistically confirmed a main effect of the factor *Cue validity*, with F(2,18) = 28.36, p < 0.001. There was also a significant main effect of the second factor *Position of the repetition target* (speech signal vs. environmental signal), with F(1,9) = 22.53, p = 0.001. Importantly, there was no significant interaction between the two factors, with F(2,18) = 1.61, p = 0.226, indicating that the attentional modulation by the cue validity worked similarly for both streams. Planned contrasts in form of paired t-tests confirmed the expected direction of the attentional modulation effect: for speech and environmental sound targets combined, participants were significantly better in detecting the repetition targets in the valid then in the invalid cueing condition, t(9) = 7.5, p < 0.001. Participants responded significantly better also in the valid than in neutral condition, t(9) = 2.83, p = 0.02 and worse in invalid compared to the neutral condition: t(9) = −4.38, p = 0.002. Also for the speech and environmental sound stream targets separately, t-tests revealed that valid cues made participants respond faster compared to invalid cues, with t(9) = 7.13, p < 0.001 in the environmental signal and t(9) = 3.75, p = 0.005 in the speech signal. A significantly more accurate response was also found between the valid and neutral condition (i.e., facilitation), with t(9) = 2.32, p = 0.045 for the speech signal and t(9) = 2.34, p = 0.044 for the environmental signal. However, comparing the invalid versus neutral condition for the two different streams (i.e. suppression effects) gave a significant better response accuracy only for the environment signal, with t(9) = −4.07. p = 0.003, but not for the speech signal, with t(9) = −1.83, p = 0.1.

Comparing the detection accuracy under valid cueing conditions for speech signals versus environmental sound signals, a paired t-test revealed that it was a bit harder to detect embedded repetition targets in the environmental signal then in the speech signal, with t(9) = 3.273, p = 0.010.

#### Reaction times

A data analysis similar to the one performed for accuracy was also conducted on reaction times, revealing congruent effects. The numeric values of the average reaction time performance in the six experimental conditions are also provided in Table 2.

**Table 2:** Experiment 2 with two overlaid speech streams.

	Valid	Neutral	Invalid
*Accuracy [%]*			
Speech	83.29 (2.67)	58.67 (2.91)	37.33 (6.34)
*Reaction time [ms]*			
Speech	754 (28)	880 (31)	931 (47)
*Sensitivity index (d’)*			
Speech	3.05 (0.24)	1.98 (0.12)	1.57 (0.15)
*Decision criterion (c)*			
Speech	0.50 (0.08)	0.77 (0.08)	1.15 (0.15)

Numeric values of the detection accuracy (in percent correct), reaction time (in ms), sensitivity indices (d’), and decision criteria (c), all across all ten participants for all three cueing conditions (valid, neutral and invalid cues), separately for the speech and environmental component of the signal. Values represent the means of each score.

A two-way repeated-measure analysis of variance (ANOVA) on mean reaction times revealed a main effect of factor *Cue validity*, F(2,18) = 8.63, p = 0.002, and a main effect of factor *Position of the repetition target* (speech signal vs. environmental signal, F(1,9) = 13.02, p = 0.005). Again, there was no significant interaction between both factors, with F(2,18) = 0.51, p = 0.610.

To investigate the direction of the observed effects, planned contrasts in form of paired t-tests were performed between the valid and invalid attention cue for speech and background, combined as well as separately. Combining data from both sound streams, participant were significantly faster identifying targets in the valid compared to the invalid cue condition, t(9) = −3.218, p = 0.010. There were also a significant differences between the valid and neutral condition, t(9) = −2.41, p = 0.039 (i.e. facilitation), and invalid and neutral conditions, t(9) = 2.44, p = 0.037 (i.e. suppression). Also for the speech and environmental sound stream targets separately, t-tests revealed that valid cues made participants respond faster compared to invalid cues, with t(9) = −3.85, p < 0.004 for targets in the environmental stream t(9) = −2.62, p = 0.028 for repetition targets hidden in the speech stream. For the environmental signal, we found evidence for facilitation effects, i.e. faster responses in the valid than in the neutral condition, with t(9) = −2.48, p = 0.035. In the speech stream, however, we did not find any significant advantage between the valid and neutral cueing condition, with t(9) = −1.83, p = 0.198. The opposite was true for suppression effects, i.e. comparing the invalid with the neutral cueing condition. Here, for the environmental signal, participants did not show any significant advantage between invalidly and neutrally cued trials, with t(9) = 1.70, p = 0.123. Instead, participants were faster in the neutral condition if the repetition was in the speech stream, with t(9) = 2.55, p = 0.03. Finally, comparing the detection accuracy under valid cueing conditions for speech signals versus environmental sound signals, a paired t-test revealed that the repetition targets were detected faster in the speech signal then in the environmental signal, t(9) = −3.683, p = 0.005. These results of mean reaction times are therefore consistent with the analysis of the detection accuracy data.

#### Signal-detection theory (SDT) analyses

We also computed sensitivity indices (d’) using the method suggested by Macmillan and Creelman^72,73^. False alarms were detected as responses given before the presentation of the target. We first calculated sensitivity indices separately for each subject and each condition and averaged the computed values separately for each of the six conditions in our 3 × 2 factorial design (with factors *Cue validity* and *Position of the repetition target*).

Figure 2C shows the average sensitivity indices across all participants as a function of cue validity and the relative position of the repetition target. Participants were clearly more sensitive to repetition targets when they were validly cued. In comparison to the neutral cue condition, valid cues made participants more sensitive to repetition targets in both the speech and environmental noise stream. Invalid cues had the opposite effect, hindering subjects’ sensitivity to those subtle auditory targets (see also Table 1 for an overview of the numeric values of d’ sensitivity and criterion. Therefore, the signal detection sensitivity analysis results were congruent with both the accuracy and reaction time data.

A two-way repeated-measures analysis of variance (ANOVA) of the sensitivity scores statistically confirmed a main effect of the factor *Cue validity*, with F(2,18) = 21.3, p < 0.001. There was also a significant main effect of the factor *Position of the repetition target* (speech signal vs. environmental signal), with F(1,9) = 23.73, p < 0.001. There was no significant interaction between the two factors, with F(2,18) =  = 0.78, p = 0.47, indicating that the attentional modulation by the cue validity worked similarly for both streams. Planned contrasts in form of paired t-tests confirmed the expected direction of the attentional modulation effect: for speech and environmental sound targets combined, participants were more sensitive to repetition targets in the valid then in the invalid cueing condition, t(9) = 7.19, p < 0.001. Comparing the valid and invalid condition with the neutral condition a significant effect of facilitation was detected for the valid condition, with t(9) = 2.98, p = 0.02 and an suppression effect was found for the invalid condition, with t(9) = −3.39, p = 0.008. Also for the speech and environmental sound stream targets separately, t-tests revealed that valid cues made participants more sensitive than invalid cues, with t(9) = 6.19, p < 0.001 and t(9) = 5.53, p < 0.001 in the environmental signal and in the speech signal, respectively. Regarding the environmental signal, validly cued trials were not significantly different from trial with neutral cues, with t(9) = 1.83, p = 0.1, but there was a facilitation of sensitivity for the speech signal, with t(9) = 2.94, p = 0.02. An opposite pattern was observed comparing the invalid condition with the neutral one, revealing a significant difference when the target was in the environmental signal, with t(9) = −2.63, p = 0.03, but no significant difference for targets in the speech stream, with t(9) = −1.80, p = 0.11. Comparing the sensitivity under valid cueing conditions for speech signals versus environmental sound signals, a paired t-test revealed that sensitivity was in general higher for the speech signals compared to environmental noise signals, with t(9) = 4.56, p = 0.001.

Figure 2D shows the average criterion (c) indices as a function of the factors *Cue validity* and *Position of the repetition target*. Participants have a similar bias and relatively liberal response criterion in the valid cueing conditions for both the speech and the environmental stream. They become more conservative in the invalid cueing condition especially when the target was embedded in the environmental signal.

A two way repeated-measures analysis of variance of the criterion scores confirmed a main effect of the factor *Cue validity*, with F(2,18) = 18.17, p < 0.001, and of the factor *Position of the repetition target*, with F(2,18) = 18.09, p = 0.002. There was also a significant interaction between the two factors, with F(2,18) = 4.48, p = 0.03. Planned paired t-tests were conducted to test the direction of the observed effects. In general there was a significantly more liberal decision criterion in the valid than in the invalid cueing condition, with t(9) = −5.70, p < 0.001. The difference in response criterion was also significant between the invalid and neutral condition, with t(9) = 4.0, p = 0.003, but not between the valid and neutral conditions, with t(9) = −0.804, p = 0.44. Interesting, for the speech and environmental signal stream separately, there was a significant liberalization of the response criterion for the environmental signal (i.e. contrasting the valid versus invalid cue condition, with t(9) = −4.86, p = 0.001), and a more conservative answering scheme when comparing the invalid and neutral condition, with t(9) = 3.87, p = 0.004. In any other contrast no significant differences were observed.

### Ethical approval and informed consent

All experiments of this study were performed in accordance with relevant guidelines and regulations and approved of by the responsible institutional review board and the University of Trento Ethical Committee on the Use of Humans as Experimental Subjects. All participants provided written, informed consent in accordance with the University of Trento Ethical Committee on the Use of Humans as Experimental Subjects. All methods were carried out in accordance with the relevant guidelines and regulations.

## Experiment 2: Attentional Weighting of Two Competing Speech Streams

In Experiment 1, we used an ecologically valid scenario of a speech signal being overlaid with environmental noise and asked participants to tune their attention to track one or the other input stream. Importantly, we equaled the low-level rhythmicity and the signal envelope, however, there exists the possibility that some low-level differences remained between the two types of stimuli and that any attentional weighting was based only on such subtle differences alone. Maybe participants could have done the task in Experiment 1 by focusing on lower-level feature instead.

Therefore, we address the question of object-based attention in a second experiment in which we present two overlaid sound streams from only one category (speech) that largely match in all low-level properties and thus require participants to fully attend to the higher-level properties. In Experiment 2, we therefore employ the same object-based repetition detection task as in Experiment 1, but have people attend one voice among other voices (both streams again overlaid spatially and temporally congruent), i.e., a listening scenario that is more similar to the classic cocktail party problem but without spatial separability of the signal sources.

### Participants

Ten participants (6 females, 4 males, mean age 27.5 years, range 25–33 years, all of them right-handed and with normal hearing) took part in Experiment 2. They all were naïve in respect to the purpose of the study, and none of them had participated in Experiment 1. They were not familiar with any of the languages used to create the speech stimuli. All participants provided written, informed consent in accordance with the University of Trento Ethical Committee on the Use of Humans as Experimental Subjects.

### Stimuli

#### Speech sound signals and overlay

In Experiment 2 we presented auditory scenes that consisted of two overlapping streams of speech conversation. There were no further embedded environmental sounds. The speech signals overlaid here were the same speech signals used also in Experiment 1. Again, a repetition segment of 750 ms was randomly embedded in either one of them, serving as a repetition target that had to be detected as fast and as accurately as possible. Both speech signals were presented from the same central position, making it impossible to use spatial information to solve the task. A set of the experimental stimuli can be freely downloaded at 10.5281/zenodo.1491058.

#### Trial Sequence and Experimental Design

As in the previous experiment we had three cueing conditions, i.e. valid, neutral, and invalid cues. Cue validity was 70%, 20% of cues were invalid, and another 10% neutral. To direct the participants’ selective attention towards one or the other speech stream, we used an auditory cue, which consisted of the first 1.0 s segment of the isolated speech signal of one of the two speakers.

A typical trial sequence in Experiment 2 is very similar to the first experiment, but now the attention was cued to one of two speech streams by a short acoustic cue, which consisted of a short pre-play segment of one of the voices. At the beginning of each trial, a fixation-cross appeared and subjects were instructed to keep central eye fixation throughout the trial. After a random interval of 1.0–1.5 s, the auditory cue was presented, directing auditory attention to one of the two speakers. In trials with neutral cue condition, no cue was given at all. After another jittered interval of 1.0–1.5 s, the combined audio scene with both overlapping speech streams started playing and continued for 5.0 s. The participants were instructed to pay attention to the cued stream and to respond with a button press as fast and as accurately as they recognized any repetition segments. Accuracy and speed were equally emphasized during the instruction.

Before the actual data collection, participants were first familiarized with the repetition segments by listen to ten individual example presentations and then performing one short sample block of 20 overlaid sound scenes in order to practice their responses to repetitions. Each subsequent testing block consisted of 60 trials. Each participant performed five experimental blocks, resulting in 300 experimental trials in total. In this second experiment, only the factor *Cue validity (*with the three conditions valid, neutral, and invalid) was relevant for the behavioral analyses. All conditions were trial-wise intermixed.

#### Data analysis

For each condition of the factor *Cue validity*, the mean and standard error of the mean (SEM) were calculated both for reaction times, response accuracies, and sensitivity indices. For the purpose of inferential statistics, repeated-measurement analyses of variance were computed on all those three behavioral measures. To further investigate systematic differences between individual conditions we computed planned contrasts in form of paired-samples t-tests between the repetition detection rates, reaction times and sensitivity scores in the valid versus invalid cueing condition.

### Results

#### Accuracy

**Figure 3:**
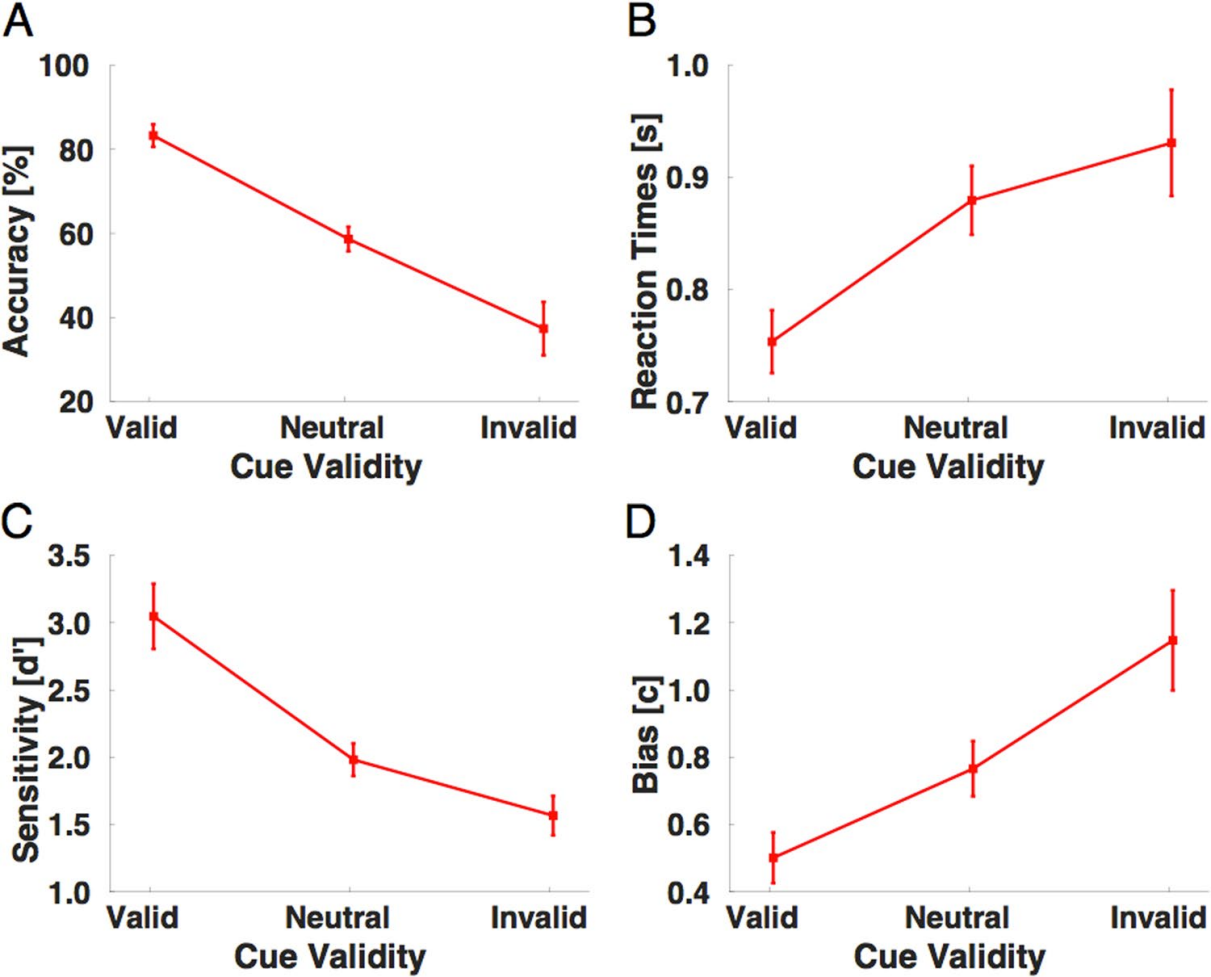
Experimental results in the repetition detection task of Experiment 2 with two overlaid speech streams. (**A**) Detection performances (in percent correct) as a function of cue validity (valid, neutral and invalid cueing condition), for the second experiment with two speech sounds. The data are shown as means and SEM. **(B**) Reaction times as a function of cue validity (valid, neutral and invalid cueing condition), for the second experiment with two speech sounds. The data are shown as means and SEM. **(C)** Sensitivity scores (d’) and decision criteria **(D)** as a function of cue validity (valid, neutral and invalid cueing condition) for the second experiment with two speech sounds. The data are shown as means and SEM.

Also in Experiment 2 with two competing speech signals, the repetition targets were detected well above chance, Fig. 3A shows the average detection accuracy, and Fig. 3B shows the corresponding reaction times with which the responses were given. There was a cue-validity effect in the sense that valid cues helped the participants in better detecting repetition targets and also speeded up their responses. Invalid cues, however, had a hindering effect compared to neutral cues (see Table 2 for all the numeric values of mean detection accuracy and reaction times).

A one-way repeated-measures analysis of variance (ANOVA) on the mean detection accuracy statistically confirmed a main effect of the factor *Cue validity*, with F(2,18) = 27.27, p < 0.001. We also calculated planned contrasts in form of paired t-tests to confirm the direction of the attentional modulation effect: participants were significantly better in detecting repetition targets in the valid then in the invalid cueing condition, t(9) = 5.44, p < 0.001 and then in the neutral cueing condition, with t(9) = 5.18, p < 0.001. Also a paired t-test comparison between invalid cueing condition and neutral cueing condition revealed a significant better accuracy in detecting the target in the neutral condition, with t(9) = −4.48 p = 0.001.

#### Reaction times

An analysis of the response times revealed congruent cue-validity effects. The numeric values of the average reaction time performance in the six experimental conditions are provided in Table 2. A one-way repeated-measure analysis of variance (ANOVA) on mean reaction times revealed a main effect of factor *Cue validity*, F(2,18) = 19.25, p < 0.001. To investigate the direction of the observed effects, planned contrasts in form of paired t-tests were performed between the valid, invalid and neutral cuing conditions. Participant were significantly faster identifying targets in the valid compared to the invalid cue condition, with t(9) = −5.25, p = 0.001, and compared to the neutral cue, with t(9) = −5.34, p = 0.001. A paired t-test between invalid and neutral condition revealed no significant effects, with t(9) = 1.7 p = 0.12. These cue-validity effects on mean reaction times are therefore consistent with the analysis of the detection accuracy data.

#### Signal-detection theory (SDT) analyses

False alarms were detected as responses given before the presentation of the target. We first calculated sensitivity indices separately for each subject and each condition and only then averaged the computed values in each of the three cueing conditions. Figure 3C shows the sensitivity scores (d’). Participants became more sensitive to the subtle repetition targets when they were validly cued. Invalid cues, however, were distracting attention and decreased sensibility to repetition targets (see Table 2 for an overview of the numeric values of sensitivity indices and criteria. Overall, the sensitivity analyses revealed congruent effects with the accuracy and reaction time data.

A one-way repeated-measures analysis of variance (ANOVA) on the sensitivity scores statistically confirmed a main effect of the factor Cue validity, with F(2,18) = 16.06, p < 0.001. Planned contrasts in form of paired t-tests confirmed the expected direction of the attentional modulation effect: participants were significantly more sensitive to repetition targets in the valid then in the invalid cueing condition, with t(9) = 4.56, p = 0.002, and also compared to the neutral cueing condition, with t(9) = 4.04, p = 0.003. No significant effect was found in a paired t-test between invalid and neutral condition: t(9) = −2.05, p = 0.07.

Again we also calculated measures of the response criterion (c) to better characterize the response bias used by the participants between the conditions. Figure 3D shows the change in the response criterion between the three conditions, with a more liberal criterion in the validly cued trials and a more conservative response bias for the invalidly cued trials (both in respect to the neutral condition, which is in the middle).

A one-way repeated-measure analysis of variance (ANOVA) on the response bias scores revealed a statistically significant effect of the factor *Cue validity*, with F(2,18) = 16.30, p < 0.001. Here, planned contrast in the form of paired t-test confirmed a significantly more liberal bias in the valid cueing condition compared to the invalid cueing condition, with t(9) = −4.71, p = 0.001 but also when comparing the valid cue condition with the neutral cueing condition, t(9) = −2.84, p = 0.02. Response biases were significantly more conservative in the invalid cueing condition compared to the neutral cueing condition, with t(9) = 3.62, p = 0.006.

### Discussion

For the present study we used novel sets of stimuli and a new repetition detection task to study object-based attention in the auditory domain. Our paradigm and stimuli were specifically conceived to tackle high-level, object-based mechanisms of selective voluntary attention, in analogy to attentional weighting paradigms used in the visual domain^5,68^. By presenting two spatially and temporally overlapping auditory scenes we were able to overcome some shortcomings of previously used dichotic listening paradigms^2,74^ regarding the role of spatial information. In classical dichotic listening tasks, participants often listen to two temporally overlapping soundscapes, attending one or the other, and it has been shown that the ability to focus attention to one particular stream depends on certain acoustic factors such as space separation, frequency distance, or semantic level of representation^75^. However, in classical dichotic listening experiments the two streams are often *spatially* separable from each other because they are typically presented to the left vs. right ear, respectively. This introduces potential confounds between high-level, e.g., object-based or semantic processes and spatial attention processes. Notably, other recent studies have also addressed the problem of object formation and selective attention without using dichotic stimulation paradigms^11,63,75–81^.

Some of the more recent neuroimaging studies made also use of modified diotic paradigms (i.e. binaural listening) in which the *same* signal is presented to both ears. In these studies participants selectively listened to one of the superimposed speech streams forming a multi-talker auditory scene^18,64,82^ or speech in synthetic noise^58,59^, or tone rhythms^66,83,84^.

An important difference to these previous studies is that we combined in Experiment 1 two acoustic streams, a *speech- and non-speech signal*, in an ecologically valid way, as it is a typical scenario in many everyday situations. Combining these different types of streams also brings advantages for the parsing of the auditory scene in the sense that both streams are less likely to be confused. In order to make the two overlaid acoustic streams comparable we introduce a procedure that allows us to equalize the envelope modulation, i.e. their coarse temporal dynamics, between them by extracting the analytic envelope from one type of signal and (across different trials) re-applying the extracted envelopes to the other type of signals. By this envelope equalization process, the two signals became very comparative in their overall temporal structure, which allowed us to directly compare them within the same attentional weighting experiment. Although, we made the two auditory streams in Experiment 1 as comparable as possible, e.g. by adjusting their respective rhythmicity and their signal envelopes, some differences in difficulty remained between the speech versus environmental noise signal. This is most likely due to the fact that the human auditory system is very well tuned to processing human speech signals, resulting in inherent behavioral advantages for identifying targets in the speech stream^85–87^. Importantly, however, Experiment 2 demonstrated that the object-based attention effects could also be observed in a listening scenario in which two very similar speech streams are overlaid. In this way the second experiment controls for both spatial and low-level feature-based attention (for features such as pitch or frequency), which both cannot be helpful in this specific task. Therefore, while the overlay of two different types of auditory streams clearly has advantages for the parsing of the scene, this is not a prerequisite for object-based attention to work.

Our approach of using a *repetition detection* task adds an important new behavioral variant to the set of diotic tasks to study selective auditory attention. Similar repetition detection tasks are often found in working memory studies^14,88^. Here the repetition detection task is implemented to study high-level object-based attention and was therefore based on a rather long integration window of 750 ms segments. In order to identify the repeating pattern in the auditory stream both segments have to be processed to a relatively deep stage, presumably to a level at which auditory objects are formed and recognized and at least to some degree attributed some semantic interpretation. This is analog to recent studies in the visual domain^68,89^ where object-based attention was studied by having participants attend to either a visual stream of spatially overlapping face and house stimuli. In this visual version of object-based attention task, subjects, too, had to identify 1-back repeats in the respectively attended stream, i.e. the re-occurrence of the same face token or house token in two successive presentation cycles. Similar to our present stimuli in the auditory domain, the argument has been made that such a repetition task is logically only possible if the face stimuli have been analyzed at least to the level of face identification processes, which are known to involve comparably late stages of the visual hierarchy in high-level visual areas concerned with object recognition. Consequently, also the attentional modulation by the task was strongest in high-level visual areas in IT cortex. Similar here in the current design, the two segments, i.e. the original sound segment and it’s repetition about 1 s later have to be processed to a comparatively high level of sound recognition, at which at least some meaning or interpretation has been computed from the segments, in order to successfully compare them. Similar to the visual variant of the task, low-level features like the pitch or spectra characteristics of an individual sound, will not allow for a successful comparison and render the detection of a segment repetition very difficult. To accomplish this also from a technical point of view, we put special care in the cutting and clipping process involved in designing the stimulus material for this repetition recognition task: In order not to leave any clipping artifacts or other detectable low-level features in acoustic sequence that could be exploited as low-level, acoustic cues for the to-be-detected repetition targets, we employed special amplitude cross-fading techniques that render the original cutting positions and transition between subsequent segments unnoticeable.

With these carefully designed stimuli and our repetition detection task, we tested mechanisms of selective attentional modulation and the effects of auditory attention on concurrent auditory streams. Following the biased competition theory^5^, selective attention is the central mechanism that biases processing of perceptual stimuli by facilitating the processing of important information and - at the same time - filtering out irrelevant information. In the present study, top-down attention to any of the two acoustic streams (i.e., the speech stream versus the environmental stream in Experiment 1 or either one of the speech streams in Experiment 2) was hypothesized to facilitate the behavioral performance in a high-level, object-based target detection task.

In both experiments, our results clearly showed the hypothesized cue validity effect: the faster and more accurate responses that were given to targets after a valid cue indicate a significant facilitation effect by top-down auditory attention. At the same time, we were also able to see significant inhibition of the respectively non-attended stream (invalid cueing condition) in comparison to a third, neutral attentional condition, in which no cue was presented at all. This replicates many previous cue-validity results and is indicative for the notion that attentional weighting works in a very similar way also on high-level, object-based auditory stimuli, presumably relying on the very same mechanisms as in other modalities or stimulus domains. Both the reaction time data and detection accuracy showed the very same pattern of results and complemented each other. Moreover analyses of signal detection sensitivity revealed congruent effects with the previous two measures: significantly higher d’ sensitivity indices can be observed in the valid cueing condition compared to both the neutral and the invalid cueing condition. There was also a tendency to adjust the decision criterion for the cued versus un-cued auditory stream. Decision criteria were more liberal for the cued and more conservative for the un-cued auditory stream, as it is typical also in classic Posner-type cueing paradigms, for example in vision^90^. These tendencies were present in both experiments, accompanying the attentional effects on perceptual sensitivity (d’). They can be explained by the fact that the experimental manipulation of the cue validity requires unequal number of trials in the valid versus invalid (versus neutral) condition, which changes the a-priori probabilities, with which the target occurs in either stream. Apparently, participants can adopt independent decision criteria (i.e., adjust their response thresholds) for parts of the auditory stream that are more or less likely to contain the target. Since we designed the experiment with a cue validity of 70%, participants may have also adopted a response strategy of being more liberal in identifying a repetition target in the cued stream, and thus also producing more false alarms than in the invalid cueing condition. The lower the probability with which a repetition target can occur in each of the competing sound streams, the more sensory evidence is required for a decision to report that repetition (and vice versa)^90^.

Comparable top-down cueing effects to ours were observed behaviorally in tasks based on the Posner-cueing paradigm^21,23,48,91–96^. In a prototypical Posner-cueing paradigm, participants have to fixate a central point on the screen and to attend covertly to either side of the fixation point in order to detect the temporal onset of a brief target stimulus. Such Posner-cueing paradigms also exist for other, non-spatial attentional scenarios such as visual features^30,32–37,39,97^, auditory features^62,65,98–103^ and visual objects^68,104–106^, all of which exhibit reliable attentional facilitation effects. The robust finding of such ‘cue-validity effects’ in our study proofs that the concept of attentional weighting and biased competition also hold for high-level attention sets in the auditory domain and that the cueing paradigm in combination with a high-level repetition detection task can be used to study attentional facilitation on an object-based level of the auditory processing hierarchy.

The process of constructing auditory objects within the auditory processing hierarchy is complex, but clearly the formation of *auditory* objects has an inherent temporal dimension, which visual objects don’t necessarily have: in audition, we store representation of certain spectro-temporal regularities only with the unfolding of the sounds over time, and on that basis we can then parse the complex auditory scene into discrete object representations^107,108^. In our present task the repetition of a fairly large temporal segment needed to be detected, which was only possible if the participants had their attention directed to the respective stream, allowing for a deep enough processing in order to build up and register condensed information in form of auditory objects, which could then be efficiently compared and matched across subsequent segments of the stream. Of course, working memory plays a crucial role in solving this kind of repetition detection task. As Conway and colleagues pointed out, auditory working memory poses important constraints on the process of object formation and the involved high-level selection processes^109,110^. Given that the temporal dimension of auditory signals is so inherently important for the parsing of object information, working memory is needed as a key component. In our task for example, in order to detect repetitions in one stream, the parsed high-level object information needs to be stored and continuously updated in a working memory buffer so that any new incoming information can sequentially be matched against these stored templates.

In conclusion, our present study complements previous research that used behavioral paradigms to investigate high-level auditory attention, e.g., in a multi-talker cocktail-party sound scenes, offering two novel aspects compared to the previous literature. First, we combined a modified Posner-paradigm and a repetition detection task in order to study the high-level, object-based aspects of selective attention in acoustic scenes. This attention task has the advantage that it cannot be solved based on the detection of simple low-level features, but instead it strictly requires a deep, object-level or semantic-level processing of the auditory stream, allowing for investigation of the attentional weighting at higher levels of the auditory processing hierarchy. Second, we used speech streams in combination with field-recordings of environmental sounds as competing sound objects, allowing us to study a particularly ecologically valid situation of competing, spatially overlapping soundscapes. Our results show robust cue-validity effects of object-based auditory attention.

## Data Availability

All datasets generated and analyzed during the current study are available from the corresponding author upon reasonable request.
